# Assessing Diversity, *Plasmodium* Infection and Blood Meal Sources in Mosquitoes (Diptera: Culicidae) from a Brazilian Zoological Park with Avian Malaria Transmission

**DOI:** 10.3390/insects12030215

**Published:** 2021-03-03

**Authors:** Lilian de Oliveira Guimarães, Roseli França Simões, Carolina Romeiro Fernandes Chagas, Regiane Maria Tironi de Menezes, Fabiana Santos Silva, Eliana Ferreira Monteiro, Marcia Moreira Holcman, Miklos Maximiliano Bajay, Adriano Pinter, Vera Lucia Fonseca de Camargo-Neves, Karin Kirchgatter

**Affiliations:** 1 Superintendence for Endemic Disease Control, SUCEN, São Paulo SP 01027-000, Brazil; lilianguima@gmail.com (L.d.O.G.); rmtironi@gmail.com (R.M.T.d.M.); fabinss30@gmail.com (F.S.S.); marciaholcman@gmail.com (M.M.H.); adrianopinter@gmail.com (A.P.); vlfcn@hotmail.com (V.L.F.d.C.-N.); 2 Institute of Tropical Medicine, School of Medicine, University of São Paulo, São Paulo SP 05403-000, Brazil; rosefs@usp.br (R.F.S.); elianafmonteiro@usp.br (E.F.M.); 3 Nature Research Centre, 08412 Vilnius, Lithuania; crfchagas@gmail.com; 4 Applied Research Department, Zoological Park Foundation, São Paulo SP 04301-905, Brazil; 5 State University of Santa Catarina, Laguna SC 88790-000, Brazil; mmbajay@gmail.com

**Keywords:** *Aedes*, captive, *Culex*, haemosporidian parasites, vectors, wildlife

## Abstract

**Simple Summary:**

Zoological gardens in forest areas host a large diversity of vertebrate species (exotic and indigenous, free-living and captive, migrant and resident), resulting in an artificial proximity of animal species that would never share the same environment in natural conditions. The presence of mosquitoes enables the transmission of vector-borne pathogens, as is the case with avian malaria parasites. The mild infections in some bird species may become a serious threat to others that do not possess a natural resistance. Thus, the identification of the potential vectors of these parasites is important for future control of these agents, aiming at the bird species conservation. In this study, we collected over 2000 mosquitoes in São Paulo Zoo and analyzed them through molecular methods. Six different mosquito species yielded positive for the targeted parasite DNA. We demonstrated that these culicids had fed mainly on bird species and we reported three mosquito species that have never been previously incriminated as potential vectors of these parasites, enabling the use of more specific measures for vigilance and mosquito control.

**Abstract:**

Avian malaria parasites are widespread parasites transmitted by Culicidae insects belonging to different genera. Even though several studies have been conducted recently, there is still a lack of information about potential vectors of *Plasmodium* parasites, especially in Neotropical regions. Former studies with free-living and captive animals in São Paulo Zoo showed the presence of several *Plasmodium* and *Haemoproteus* species. In 2015, a pilot study was conducted at the zoo to collect mosquitoes in order to find out (i) which species of Culicidae are present in the study area, (ii) what are their blood meal sources, and (iii) to which *Plasmodium* species might they be potential vectors. Mosquitoes were morphologically and molecularly identified. Blood meal source and haemosporidian DNA were identified using molecular protocols. A total of 25 Culicidae species were identified, and 6 of them were positive for *Plasmodium*/*Haemoproteus* DNA. Ten mosquito species had their source of blood meal identified, which were mainly birds, including some species that were positive for haemosporidian parasites in the former study mentioned. This study allowed us to expand the list of potential vectors of avian malaria parasites and to improve our knowledge of the evolutionary and ecological relationships between the highly diverse communities of birds, parasites, and vectors present at São Paulo Zoo.

## 1. Introduction

Malaria is a disease caused by infection with the protozoa of the genus *Plasmodium* (Haemosporida, Plasmodiidae). These parasites are a group of widespread heteroxenous protists that can be found parasitizing reptiles, birds, and mammals, which are transmitted by blood-sucking dipterans belonging to the Culicidae [1]. In birds, avian malaria and related haemosporidian (such as *Haemoproteus* and *Leucocytozoon*) parasites are widely used models in studies of evolutionary and ecological parasite–host interactions (rev. in [2]). Avian *Plasmodium* can be transmitted by several genera of mosquitoes (Culicidae): *Aedes*, *Anopheles*, *Armigeres*, *Coquillettidia*, *Culex*, *Culiseta*, *Lutzia*, *Mansonia*, *Psorophora*, and *Wyeomyia* [1,3,4,5,6]. However, the vectors species are still the least known subject in avian haemosporidian research, and it is here where dedicated effort is needed.

Neotropical regions are considered hotspots of avian diversity but there are still few studies focusing on bird parasites in these locations, and several new haemosporidian species are still likely to be discovered [7]. Moreover, in these regions, specifically in captive environments, parasitic diseases can be commonly found and spread easily, leading to a fatal outcome to some bird species [8,9,10]. High density of animals is common in captivity and some species can be exposed to parasites to which they may be not evolutionarily adapted and therefore have no competent immune responses [11]. Thus, zoological gardens condition allows a peculiar exposure dynamic to novel hosts (sympatric and introduced) such as among wild, captive, migratory, and non-migratory birds (rev. in [2]).

Studying avian malaria at the São Paulo Zoo in 19 different orders of free-living and captive animals, we identified a high prevalence of *Plasmodium* and *Haemoproteus* besides establishing DNA barcoding for new lineages [9,12,13]. In order to identify the potential vectors, we conducted a pilot study to capture mosquitoes, with the aim of responding to the following questions: (i) which Culicidae species are present in the study area; (ii) on which vertebrate species do these mosquitoes feed; and (iii) to which *Plasmodium* species might they be potential vectors.

## 2. Materials and Methods

### 2.1. Study Area

**Figure 1:**
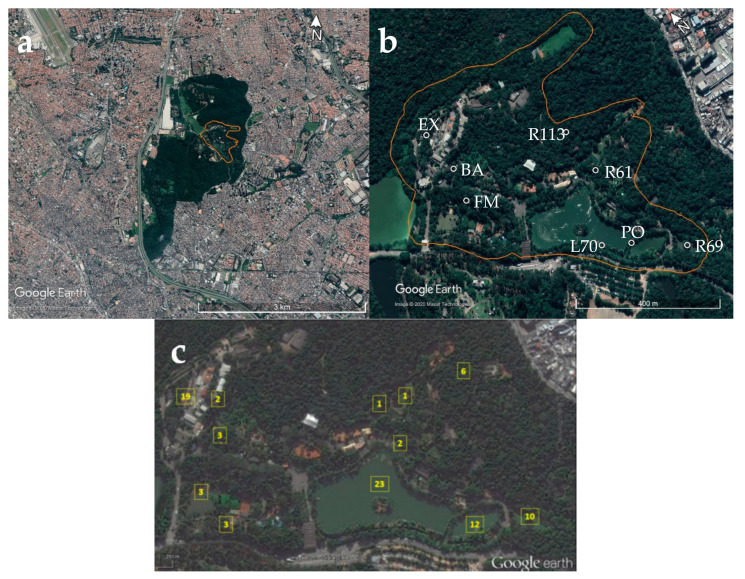
Location map of Parque Estadual das Fontes do Ipiranga (PEFI) in São Paulo, Brazil (**a**). In zoom, São Paulo Zoo and the sampling areas (white circles) (**b**) chosen considering the number of infected individuals distributed according to their probable sites of infection, after evaluation of the clinical and parasitological history of the infected birds that were analyzed in a previous study [13] (**c**).

The study was performed inside the São Paulo Zoo, which is located in the Parque Estadual das Fontes do Ipiranga (PEFI), an area of Atlantic Forest remnant with intense anthropic influence at its borders. In addition to the approximately 3000 captive animals, many free-living and migratory birds inhabit the surrounding areas of the existent lakes in the park area (Figure 1a). Eight sampling areas (Figure 1b) were selected considering the distribution of the probable sites of infection of captive animals surveyed in a previous study [13] (Figure 1c).

**Figure 2:**
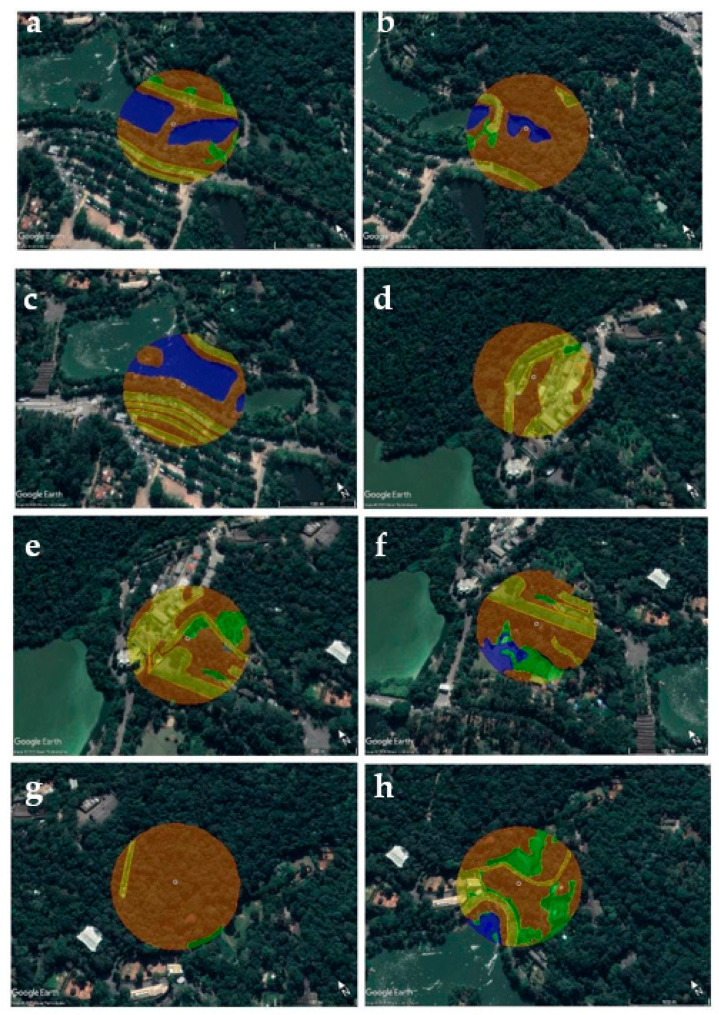
Sampling areas in São Paulo Zoo: site PO (**a**), site R69 (**b**), site L70 (**c**), site EX (**d**), site BA (**e**), site FM (**f**), site R113 (**g**), and site R61 (**h**). Small circles in the center indicate the exact point where the Center for Disease Control (CDC) traps were installed. Buffers extending 200 m diameter were created around each sampling site to define the surrounding landscape. Yellow represents areas where there is human activity (paving and edification), blue represents lake area, green shows bush/sand/grass areas, and orange shows forest cover.

As shown in Figure 2, the sampling areas were site PO (46°37′5.05″ W, 23°39′8.51″ S), located in the bridge between the two big lakes; site R69 (46°37′0.95″ W, 23°39′11.89″ S), located near an enclosure with several Anatidae species and a small lake; site L70 (46°37′7.46″ W, 23°39′6.95″ S), located on the shore of the larger lake that is close to the park boundaries; site EX (46°37′14.00″ W, 23°38′49.00″ S), the only sampling area located outside of the visitation area of the park and close to several buildings; site BA (46°37′13.97″ W, 23°38′53.00″ S), located in the visitation area of the zoo called “Bosque das Aves” (wood of birds) with several bird species; site FM (46°37′15.00″ W, 23°38′56.00″ S), located in the visitation area of the zoo near an enclosure with a big flamingo group; site R113 (46°37′3.00″ W, 23°38′57.00″ S), a visitation area where a big diversity of raptors species is kept; site R61 (46°37′3.18″ W, 23°39′1.41″ S), a visitation area with several bird species from different families (Anatidae, Anhimidae, Phoenicopteridae, and Gruidae). The location of each point was obtained using a global positioning system (GPS) (GPSMAP 62s, GARMIN, Olathe, KS, USA).

### 2.2. Mosquito Collection and Identification

Mosquitoes were collected in March of 2015, once a week for 4 weeks, using 2 different methods. First, in order to collect engorged females for blood meal source investigation, we carried out daytime collections in the morning in sites L70, BA, FM, and R113 with a Nasci aspirator [14]. Second, for the analysis of Culicidae diversity, we carried out night collections in all the 8 sites using only automatic traps (Center for Disease Control –CDC—Miniature light traps) [15] baited with CO_2_ (dry ice), placed approximately 1.5 m off the ground. The traps were set up at dawn and beginning of the night and removed about 14 h later on the following morning.

Climatic conditions were recorded for all collections using data from the weather station located inside the PEFI (Instituto de Astronomia, Geofísica e Ciências Atmosféricas de São Paulo, Universidade de São Paulo, http://www.estacao.iag.usp.br/) (accessed on 19 October 2020) (Table A1 in Appendix A).

Collected mosquitoes were killed with chloroform steam, placed in individual tubes, and stored in a styrofoam box with ice before storage in a −20 °C freezer. In the laboratory, Culicidae specimens were morphologically identified on chilled tables with a stereomicroscope using taxonomic keys [16,17,18]. After identification, the head/thorax portion was separated from the abdomen and frozen apart. Head/thorax portion was used to detect the presence of DNA of *Plasmodium*/*Haemoproteus*, and the abdomen of engorged females was used to identify blood meal sources. Both protocols are described ahead.

This study was performed according to the Ethical Principles in Animal Research and received approval by the Ethics Committee of Institute of Tropical Medicine, University of Sao Paulo (CPE-IMT/294A, 30 October 2014, and CPE-IMT/371A, 4 October 2019), and the Brazilian Ministry of Environment (SISBIO 34605-7, 27 October 2016).

### 2.3. Plasmodium/Haemoproteus Detection

The evaluation of the presence of *Plasmodium*/*Haemoproteus* was made only with the head/thorax portion of the female specimens, increasing the chances of detecting the DNA of parasites in the salivary gland, where the sporozoite infective stage are, indicating that they are potential vectors. Mosquitoes were separated into pools containing 1-11 specimens, according to species (morphologically identified), date, and sampling site (except Pool 103). Pools were triturated in FastPrep-96 (MP Biomedicals, Solon, OH, USA) in 1.4 mm ceramic beads (MagNA Lyser Green Beads-Roche Molecular Systems) in combination with 2 ceramic beads coated with 6.35 mm zirconium oxide (MP Biomedicals) in Master Mix lysis buffer [200 μL Nuclei Lysis Solution, 50 μL EDTA (EthyleneDiamine Tetraacetic Acid) 0.5 M (pH 8.0), 20 μL proteinase K (20 mg/mL) and 5 μL RNase A Solution] for 3 min at 1800 rpm and then centrifuged for 5 min at 14,000 rpm at room temperature. DNA extraction was then followed using the Wizard SV 96 Genomic DNA Purification System (Promega). The lysates were transferred into the columns and washed according to the manufacturer’s instructions. DNA was eluted in 100 μL of Nuclease-Free Water and stored at −20 °C until analysis.

Polymerase chain reactions (PCR) were conducted using a nested protocol targeting the mitochondrial cytochrome b (*cytb*) gene of *Haemoproteus* and *Plasmodium* species [19]. The first reaction used the primers HaemNFI and HaemNR3 and 5 µL genomic DNA. In the nested reaction, performed with a second pair of primers, HaemF and HaemR2, we used 1 µL of the product from the first reaction as a template. In each PCR, two controls were carried out in parallel: one positive control (mosquito sample with known *Plasmodium* sp. infection) and one negative control (ultrapure water without DNA).

All PCR products were evaluated by running 10 µL on 1% agarose gel. Positive samples were sequenced by BigDye Terminator v3.0 Cycle Sequencing Kit in ABI PRISM^®^ 3500 Genetic Analyzer (Applied Biosystems, Carlsbad, CA, USA) using the same primers from the nested PCR reaction (HaemF/HaemR2). *Cytb* sequences of ≈480 bp were aligned, edited, and analyzed using the BioEdit software [20]. Lineages were identified using BLAST (basic local alignment search tool) with sequences from the MalAvi database [21] in order to verify the *Plasmodium/Haemoproteus* lineages or species (if available).

### 2.4. Mosquito DNA Barcoding

Morphological identification of mosquito species was confirmed through processing all the abdomen portions of the haemosporidian-positive mosquitoes and those from the pools that were positive for these parasites. They were processed separately. The abdomens were triturated, and DNA was extracted as described in the previous section. Then, they were submitted to a protocol for molecular identification using DNA barcoding. A 710 bp fragment of the mitochondrial cytochrome C oxidase I (*cox1*) gene was amplified by PCR using universal primers (LCO1490 and HC02198) [22] and the conditions of the PCR were previously described [23]. PCR products were sequenced by BigDye Terminator v3.0 Cycle Sequencing Kit in ABI PRISM^®^ 3500 Genetic Analyzer (Applied Biosystems, Carlsbad, CA, USA) using PCR primers. Sequences were compared with other sequences deposited on the GenBank database (www.ncbi.nlm.nih.gov/blast/Blast.cgi (accessed on 19 October 2020)). The best close match (BCM) algorithm was used to identify the best barcode matches of a query, and the species name of that barcode was assigned to the query if the barcode was sufficiently similar [24]. Here, positive identifications were considered for the sequences that presented similarity > 99%.

### 2.5. Blood Meal Source Identification

The abdomen of engorged females was used in this step. The samples were processed using a PCR protocol that amplifies host DNA from the mosquito blood meal with primers L14841 and H15149 [25] that was designed to amplify fragments with ≈300 bp of the mitochondrial *cytb* gene from a wide range of animals, including mammals, birds, amphibians, reptiles, and fishes. This methodology was successfully used to identify the blood meal sources in mosquitoes from São Paulo [26], including São Paulo Zoo [11]. Amplified fragments were sequenced directly using the corresponding flanking primers. Obtained sequences were compared to other sequences deposited on GenBank database (www.ncbi.nlm.nih.gov/blast/Blast.cgi (accessed on 19 October 2020)). Positive identification and host species assignment were made by BCM as described above, and sequences were considered with positive identifications when they presented a match of >99%.

### 2.6. Landscape Data Analysis

The locations of the CDC traps were georeferenced in each of the eight selected sites and the vegetation or land use were identified by visual analysis, in loco and on high-resolution satellite images (CNES/Astrium, DigitalGlobe and Terrametrics compositions, with passage date until October 2012), provided in Google Earth Pro 7.1.5.1557 (Google, Inc., Mountain View, CA, USA). Landscape metrics were obtained in the same software.

Landscape composition and metric configurations were calculated and used as explanatory variables to investigate whether the environment may have an influence on the abundance of the culicid species. Buffers extending 200 m diameter were created around each sampling site, where CDC traps were settled, in order to define the surrounding landscape (Figure 2). The buffer size was determined in order to characterize the surrounds that might be directly influencing the sampled mosquito population and to avoid excessive overlapping buffers from different collection points. Landscape composition was measured by the software through drawn polygons over a layer composed by a proper scaled geotagged map of the São Paulo Zoo, in order to identify the pavement and edification areas, and over a satellite image layer in order to identify the different classes of the natural areas; six classes of vegetation or land use were observed: forest area edge length, forest area with high trees, lake area, edification, paving, and bush/sand/grass area (Figure 2).

### 2.7. Data Analysis

Total infection rate per mosquito species is the quotient of the number of infected specimens of a species by the total number of analyzed specimens of that species.

Mosquito sampling effort was estimated by plotting a species accumulation curve to verify the sampling sufficiency to assess richness, directly related to the number of rare species in the samples, for each of the eight collection sites, and the measures of biodiversity were obtained using Shannon–Winer Diversity Index (H), as described elsewhere [27].

We also analyzed if the mosquito diversity was affected by the landscape composition factors. A linear regression of anthropogenic changes on mosquito’s density was obtained in each site, using two classes of land use directly related to urbanization (edification and paving areas). Linear models were implemented using the *lm* function in the software R 4.0.0. [28].

## 3. Results

### 3.1. Diversity of Culicidae Species

**Figure 3:**
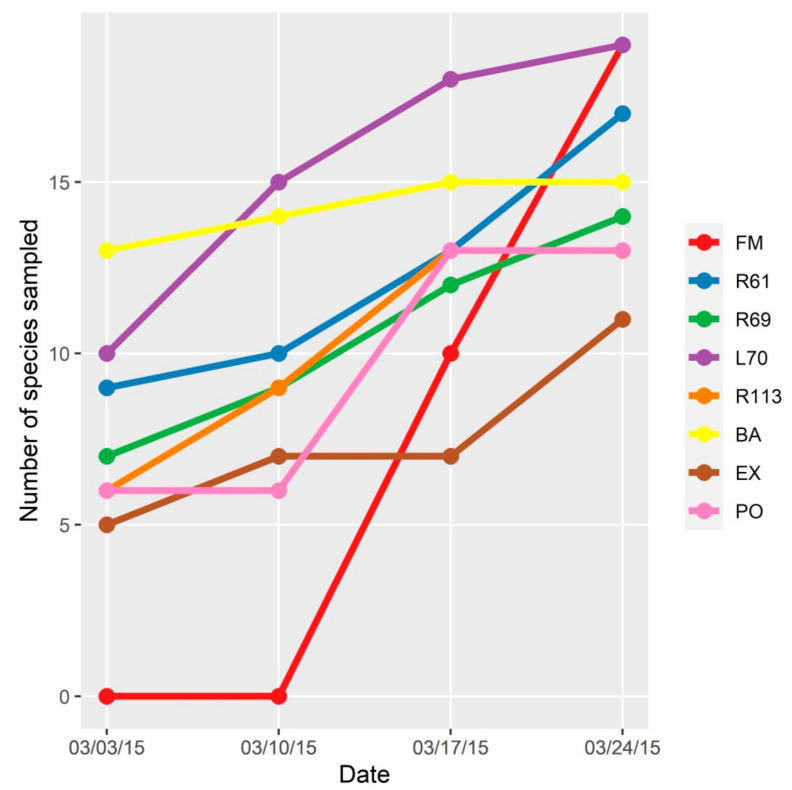
Accumulation curve of Culicidae species per sampling site in São Paulo Zoo. Absolute number of species (*Y*-axis) is shown in each point on the curve and the accumulation number of species on each week of collection (MM/DD/YY) (*X*-axis).

The accumulation curve of Culicidae species per sampling site in São Paulo Zoo, using CDC traps and Nasci aspirator, constructed using the absolute number of species by date, showed little or no variation in the number of species sampled in the weeks 3 and 4, indicating that for almost all sampling sites, the total diversity of existing species was sampled with these two methods (Figure 3).

The H index values were 2.0 for PO, 2.1 for 69, 2.3 for EX, 2.3 for R61, 2.3 for BA, 2.4 for L70, 2.4 for R113, and 2.6 for FM. The mean value was 2.33, and the median was 2.37. The classification of the areas was made following the criteria suggested by Baliton et al. [29]. Only the site FM was classified as moderate biodiversity, whereas all other seven areas fit into low biodiversity classification.

A total of 2039 female mosquitoes were collected and identified in 30 species or species groups, distributed in seven genera (*Aedes*, *Anopheles*, *Coquillettidia*, *Culex*, *Limatus*, *Mansonia*, and *Uranotaenia*) with 75.8% of mosquitoes from *Culex* genus (Table 1). In total, 760 (37.3%) specimens were identified to the subgenus level and 1279 (62.7%) to the species level. The most abundant identified species was *Aedes scapularis* (*n* = 294; 14.4%), followed by *Culex coronator* complex (*n* = 225; 11%), and *Culex bidens* (*n* = 209, 10.2%) (Table 1). From the 1545 mosquitoes identified inside the *Culex* genus, 48.5% of specimens were identified only as *Culex* (*Mel*.) Melanoconion Section or *Culex* (*Cux*.) sp. (Table 1).

Mosquito species identification based on *cox1* sequences corroborated the morphological identification for all the mosquitoes of *Aedes* genus. For mosquitos of *Mansonia* genus, the identification of species based on best close match was unsuccessful since the queries in GenBank returned with BCM far below the threshold value (≈90%). Moreover, in most of the pools, different species of a genus (or species complex) were identified, with it being impossible to determine the mosquito species that was positive.

### 3.2. Haemosporidian Diversity and Prevalence in Mosquitoes

The parasite detection was performed for 74% of collected mosquitoes (1506 specimens), aiming mainly at mosquitoes that were morphologically identified at the species level; thus, 520 *Culex* (*Cux.*) spp. specimens were excluded (Table 1). Molecular analyses revealed nine positive individual mosquitoes and four pools (Table 1). Five from nine positive individual mosquitoes were identified as *Aedes scapularis*. Parasite DNA isolated from mosquitoes belonged to different haemosporidian parasites: *Plasmodium nucleophilum* (*cytb* lineages pDENPET03), *Plasmodium* sp. (pCULMAX01, pCULEX05, and pCULEX06), and *Haemoproteus (Parahaemoproteus*) sp. (hAEDSCA01) (Table 2). The lineages pCULMAX01, pCULEX05, pCULEX06, and hAEDSCA01 are new descriptions according to the MalAvi database. Haemosporidian sequences obtained in this study were deposited in GenBank (MW492356-MW492368). Of the positives, seven individuals and one pool (with eight mosquitoes) were collected on the same day (3 March 2015), and most of them were collected in the same sampling area (BA) (Table 2).

### 3.3. Landscape Characteristics and Mosquito Diversity

With regards to landscape metrics, R113 showed higher values of forest cover and lower values of areas submitted to anthropogenic changes than other sites, while EX showed higher values of anthropogenic changes and lower values of forest cover and total edge length than other sites. Table 3 shows the landscape metrics for the areas close to each collection site.

**Figure 4:**
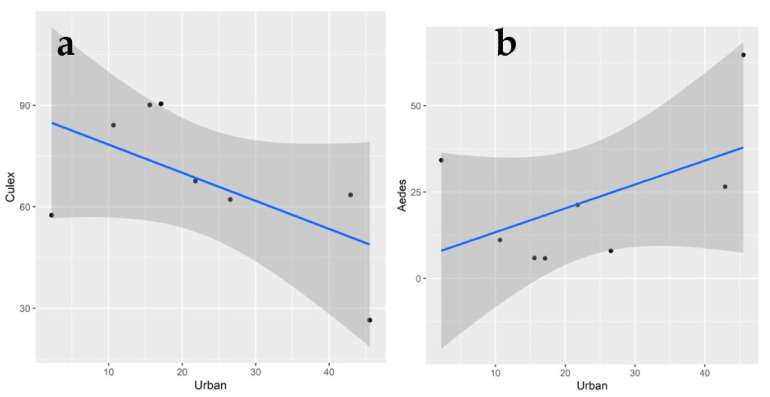
Frequency of *Culex* (**a**) and *Aedes* species; (**b**) collection and area of edification in each site in the São Paulo Zoo.

In regard to the species of mosquitoes obtained in each collection site and the respective landscape composition, we observed that in the sampling sites, the lower the level of anthropogenic changes (here called “urbanization”, including edification and paving areas) and the more water availability, the more *Culex* species were collected. On the other hand, the more that there were anthropogenic alterations, the more frequently we found that *Aedes* species were obtained. This was better observed in the site EX, which was the site with more “urbanized” area, where we collected the larger and smallest quantity of *Aedes* and *Culex*, respectively. However, although the results of the generalized linear model graphically indicate the trend, it is not supported by statistical testing (lm Urban~*Aedes: p*-value: 0.1885 and Pearson = 0.1463; lm Urban~*Culex*: *p*-value: 0.1223 and Pearson = 0.2419) (Figure 4).

### 3.4. Blood Meal Source

A total of 32 engorged mosquito females had their blood meal source successfully identified, mainly *Culex (Cux.) coronator* (5 individuals), *Culex (Cux.) declarator* (10 individuals), and *Culex (Cux*.) sp. (5 individuals) (Table 4 and Table A2). All the engorged mosquito species had fed on both mammals and birds’ blood, essentially in the same proportion. Engorged specimens were collected from all collection sites, but 35.7% of them were collected at BA. From the 11 avian species identified as being the source of blood meals, 5 (45%—*Spizaetus ornatus, Cygnus atratus, Balearica regulorum, Pavo muticus*, and *Rhea americana*) are found only in captive at the zoo, 1 can be found both in captive and free-living in the area (*Pipile jacutinga*), and the other 5 species are free-living ones (*Anser anser, Ardea herodias, Nycticorax nycticorax, Cathartes melambrotus*, and *Turdus rufiventris*). All the three mammalian species detected were found to be free-living.

## 4. Discussion

Understanding the biodiversity of mosquito species and their association with anthropogenic actions and forest area is important for increasing the knowledge of the possible changes in mosquito populations and pathogen transmission [30,31]. The diversity of avian haemosporidian vectors remains poorly studied in the Neotropical region and, more specifically, in the Atlantic Forest. Here, we collected mosquitoes during March 2015, weekly, in eight different locations that include the areas of probable *Plasmodium/Haemoproteus* infection site of birds from a previous study [13]. March was chosen because it is the end of summer/beginning of autumn in the southern hemisphere. Previous studies showed that although the number of mosquitoes caught in the summer is higher, it is in the autumn that the positivity for hemosporidian parasites reaches its peak [32]. The H index showed that the Culicidae biodiversity in the studied area was classified as low, which is explained for the higher abundance of a few mosquito species, such as *Aedes scapularis* and *Culex* spp. These species groups are known as important vectors for several arbovirus and other parasites and, in combination with the presence of several resident and migratory bird species, highlights the studied area as a potential and noteworthy entry for emerging zoonotic diseases in the city of São Paulo.

A big portion (three-quarters) of collected mosquitoes in this study identified as *Culex* genus can be a biased by the method and overnight period of capture. Additionally, although not statistically significant, it was visually possible to identify in the graphs as different in terms of the mosquito species prevalence, depending on the sampling site, with a possible tendency to observe more *Culex* species in areas with less anthropogenic changes. Here, five species were identified as being potential vectors of avian *Plasmodium* parasites, three of them belonging to the *Culex* genus. These findings, together with the high degree of ornithophilic feeding preference found in *Culex* mosquitoes from São Paulo Zoo, support the idea that this must be the main genus of vectors of these parasites for birds in the area, as has been demonstrated in other regions [33,34].

It is important to note that in order to analyze if a Culicidae species is a competent *Plasmodium* vector, it is necessary that sporozoites, the infective stages of haemosporidian parasites, are detected in the insect salivary glands [5], for which investigation requires insect dissection and slide preparation [35]. Thus, the presence of parasite DNA in salivary glands or in head/thorax portions should be carefully analyzed. In the present study, 13 samples belonging to 5 species (*Aedes (Stg.) albopictus*, *Aedes (Och.) scapularis*, *Culex (Cux.) maxi*, *Culex (Cux.) nigripalpus*, and *Culex (Cux.) coronator* complex) and two subgenera without species identification (*Mansonia (Man.)* sp. and *Culex (Cux.)* sp.) were PCR-positive for *Plasmodium*/*Haemoproteus*. Few species of mosquitoes are currently considered competent for the transmission of *Plasmodium* in birds, with *Aedes*, *Anopheles*, *Culex*, and *Culiseta* serving as the most important vectors [1,3,6]. Here, from the five species found positive, only *Aedes albopictus* and *Culex (Cux.) nigripalpus* had already been reported as harboring haemosporidian infection in the literature (see [6,36]). Thus, in this study, we expanded the list of potential vectors of avian malaria, with the first detection of *Plasmodium* parasite DNA in *Aedes scapularis*, *Culex (Cux.) maxi*, and *Culex coronator* complex.

The occurrence of avian *Plasmodium* lineages in *Mansonia* mosquitoes from Brazil has been reported in two species (*Mansonia titillans* and *Mansonia pseudotitillans*) [37]. Thus, it is likely that *Mansonia* species play a role in avian malaria transmission in this country, but additional studies are necessary to confirm this hypothesis, as well as the species involved in this process. Moreover, about our finding of *Haemoproteus (Parahaemoproteus*) DNA in *Mansonia* (*Man.*) sp. and *Aedes scapularis*, it is important to note that these parasites are transmitted by biting midges of the genus *Culicoides* (Ceratopogonidae), and thus not by mosquitoes [1,6]. *Parahaemoproteus* is the most diverse group of avian haemosporidian parasites [1,21], but the vector species have only been determined for a small number of parasites (see [6,38]). Although the presence of *Haemoproteus* DNA in Culicidae has already been reported [4,36,39,40], this does not mean that these insects can play a role in its transmission. Experimentally exposure to *Haemoproteus*-infected birds demonstrated that these parasites are unable to develop in mosquitoes [40,41]. One of them reported the abortive development of two *Haemoproteus* species in the wild mosquito *Ochlerotatus cantans*, showing that oocyst development occurs in the entire digestive tract of the mosquito (head, thorax, and abdomen), resulting in the presence of parasite DNA that can serve as a template in PCR-based protocols [40]. Moreover, the saliva of mosquitoes exposed to birds infected by *Plasmodium* and *Plasmodium/Haemoproteus* was analyzed, and only *Plasmodium* DNA was found, confirming that Culicidae insects do not support sporogonic development of *Haemoproteus* parasites [41]. This highlights the importance of combining morphological and molecular protocols in vector studies [42].

*Plasmodium nucleophilum* (pDENPET03) was the main parasite lineage detected in the present study, showing that several species of mosquitoes can transmit it. This *Plasmodium* species was detected in former studies conducted by our group in the study site, being one of the most common lineages reported [9,13]. Combining the results of the present study about the Culicidae vector occurrence and former studies with haemosporidian in captive birds, we were able to conclude that pDENPET03 is the most common lineage circulating in the São Paulo Zoo area. Moreover, the new lineage pCULMAX01, detected on *Culex (Cux.) maxi,* presented 99% of identity with pDENPET03 (just one nucleotide substitution) and it likely belongs to *P. nucleophilum*, but morphological investigation with positive blood smears is necessary to confirm this species.

The lineage pVIOLI03 was detected here in *Culex (Cux.) nigripalpus*, but it was not detected in birds in the former studies [9,12,13]. This can be explained due to the fact that this lineage infects mainly Passeriformes, and this group had few individuals sampled before, although there is a high diversity in the area (mainly from Turdidae) [43]. In fact, the lineage pVIOLI03 has also been reported in passerines from Vireonidae and Trochilidae in the Brazilian Atlantic Forest [44].

The lineages pCULEX05 and pCULEX06 are new records in the MalAvi database. However, they presented 99% of identity with pTUMIG03, a lineage of *Plasmodium unalis.* This parasite was described in Colombia in Turdidae [45], and even though that this parasite has been reported mainly in this family of passerines, including in Brazilian Atlantic Forest [44], it can also infect birds from other families in South and North America (see the MalAvi database). Moreover, pTUMIG03 was also found in *Mansonia pseudotitillans* in the Brazilian dry tropical forest (Caatinga) [37]. Further morphological studies with blood smears are necessary to confirm this parasite species.

The lineage hAEDSCA01 is a new *Haemoproteus (Parahaemoproteus*) sp. lineage in the MalAvi database and it is closely related to hELAALB01, which has been reported in the Brazilian Atlantic Forest in *Elaenia albiceps*, a migratory species of Tyrannidae passerines [44]. The lineage hMYISWA01, also *Haemoproteus (Parahaemoproteus*) sp., was found in this study and has already been reported in Brazil [46,47], but in another two Brazilian biomes: savanna (Cerrado) and dry tropical forest (Caatinga). Thus, with these findings, we are expanding the occurrence area of this haemosporidian lineage in Brazilian territory.

For the blood meal source, one of the sequences detected in engorged females had 99% identity with a sequence from the great blue heron *Ardea herodias* (GenBank #AY509650), a bird from the Ardeidae family with geographical distribution in Central and North America [48]. In an avifauna survey recently conducted in the study site, this bird species was not reported, but *Ardea cocoi* and *Ardea alba* were found [43]. A similar situation can be seen in the sequence of the greater yellow-headed vulture *Cathartes melambrotus* detected in *Culex (Cux.) declarator.* This Cathartidae species occurs in the Amazon region, not being reported in São Paulo city. At the time that the mosquitoes were collected, the São Paulo Zoo did not keep any of these species in captivity. The avifauna survey reported the presence of *Coragypus atractus* [43]. These situations can be explained by the fact that not all Brazilian bird species have *cytb* sequence records deposited in the GenBank database, making it difficult to obtain precise identification of species by the BCM method.

It is worth mentioning that DNA of the black-fronted piping guan *Pipile jacutinga* and the green peafowl *Pavo muticus* were detected in blood meals of mosquitoes collected in site BA, where the enclosures of these birds are located. These two species of birds were found to be *Plasmodium*-positive in the past, with *P. jacutinga* harboring infections by *P. nucleophilum* pDENPET03 [13]. In the sampling sites R69 and PO, mosquitoes that had fed on the black swan *Cygnus atratus* were detected. Similarly, this was already expected, since a big group of these animals lives in the lakes. On the other hand, at the BA site, one mosquito had fed on *C. atratus* and another one had fed on the ornate hawk-eagle *Spizaetus ornatus*, both birds that are not located in this place. These findings could be useful to determine the flight range distance of these mosquitoes. However, in these and also in other situations, we faced three difficulties: (i) some birds had been moved from the visitor area for veterinary care, (ii) mosquitoes had fed on vertebrate species that were not part of the zoo collection, or (iii) an insufficient number of mosquitos to determine flight range distance with accuracy had been obtained. Thus, analyses of flight range distances in terms of the blood meal analysis were not possible.

Lastly, the presence of blood meals from the black-crowned night-heron *Nycticorax nycticorax* and of the rufous-bellied thrush *Turdus rufiventris*, free-living birds, reinforces the possibility of the parasites being transmitted between free-living and captive birds.

## 5. Conclusions

In this study, we expanded the list of potential vectors of avian malaria, with the first detections of *Plasmodium* DNA in *Aedes scapularis*, *Culex (Cux.) maxi,* and *Culex coronator* complex, and we found four new haemosporidian lineages in mosquitoes. Therefore, molecular identification of parasites in Brazilian mosquito species should be encouraged, aiming to improve the knowledge of the evolutionary and ecological relationships between the highly diverse communities of birds, parasites, and vectors present in the country and to propose future control protocols of these infections.

## Figures and Tables

**Table 1:** Diversity of Culicidae species and *Plasmodium/Haemoproteus* prevalence in mosquitoes from São Paulo Zoo. Rate of infection (%) for each mosquito species or group is given in brackets.

Mosquito Species (or Species Groups)	Total Collected	Total Analyzed	Individual Analyses	Positive Samples	Pools	Total Samples in Pools	Positive Pools
*Aedes (Och.) scapularis*	294	294	160	5 (1.7)	16	134	-
*Aedes (Stg.) albopictus*	15	15	6	1 (6.6)	2	9	-
*Anopheles (Nys.) evansae*	33	33	8	-	3	25	-
*Anopheles (Nys.) galvaoi*	3	3	3	-	-	-	-
*Anopheles (Nys.)* spp.	5	5	5	-	-	-	-
*Anopheles (Nys.) strodei*	22	22	6	-	2	16	-
*Coquilettidia (Rhy.) nigricans*	1	1	1	-	-	-	-
*Coquillettidia (Rhy.) hermanoi*	2	2	-	-	1	2	-
*Coquillettidia (Rhy.)* sp.	1	1	1	-	-	-	-
*Culex (Cux.) acharistus*	8	8	5	1 (12.5)	1	3	1 (12.5)
*Culex (Cux.) ameliae*	65	65	10	-	7	55	-
*Culex (Cux.) bidens*	209	206	21	-	20	185	-
*Culex (Cux.) chidesteri*	36	35	6	-	4	29	-
*Culex (Cux.) coronator* complex	225	221	56	-	19	165	2 (0.90)
*Culex (Cux.) declarator*	136	135	41	-	11	94	-
*Culex (Cux.) dolosus*	5	5	5	1 (20.0)	-	-	-
*Culex (Cux.) habilitator*	66	64	49	-	2	15	-
*Culex (Cux.) nigripalpus*	10	10	5	-	2	5	-
*Culex (Cux.) quinquefasciatus*	1	1	1	-	-	-	-
*Culex (Cux.) saltanensis*	2	2	-	-	1	2	-
*Culex (Cux.) scimitar*	28	28	-	-	3	28	-
*Culex (Cux.)* spp.	529	9	9	-	-	-	-
*Culex (Mcx.) imitator*	5	5	-	-	1	5	-
*Culex (Mel.)* Melanoconion Section	220	219	61	-	19	158	-
*Limatus durhamii*	1	1	1	-	-	-	-
*Mansonia (Man.) indubitans*	65	65	21	1 (1.55)	6	44	1 (1.55)
*Mansonia (Man.) pseudotitillans*	18	17	5	-	2	12	-
*Mansonia (Man.)* spp.	5	5	5	-	-	-	-
*Mansonia (Man.) titillans*	25	25	14	-	2	11	-
*Uranotaenia (Ura.) pulcherrima*	4	4	-	-	1	4	-
Total	2039	1506	505	9 (0.60)	125	1001	4 (0.26)

**Table 2:** Data of the haemosporidian positive samples.

ID Positive Sample	ID Mosquito	Species (According Table 1)	Species (According DNA Barcode) #	Collection Day/Month	Collection Site	Parasite/ Lineage/GenBank Accession
12	12	*Aedes (Stg.) albopictus*	*Aedes (Stg.) albopictus*	03/03	EX	*P. nucleophilum*/ pDENPET03/MW492356
16	16	*Culex (Cux.) acharistus*	*Culex (Cux.) coronator*	03/03	EX	*P. nucleophilum*/ pDENPET03/MW492357
44	44	*Mansonia (Man.) indubitans*	*Mansonia (Man.)* sp. †	03/03	R113	*P. nucleophilum*/ pDENPET03/MW492358
60	60	*Aedes (Och.) scapularis*	*Aedes (Och.) scapularis*	03/03	BA	*P. nucleophilum*/ pDENPET03/MW492359
67	67	*Aedes (Och.) scapularis*	*Aedes (Och.) scapularis*	03/03	BA	*P. nucleophilum*/ pDENPET03/MW492360
76	76	*Aedes (Och.) scapularis*	*Aedes (Och.) scapularis*	03/03	BA	*P. nucleophilum*/ pDENPET03/MW492361
87	87	*Aedes (Och.) scapularis*	*Aedes (Och.) scapularis*	03/03	BA	*P. nucleophilum*/ pDENPET03/MW492362
121	121	*Culex (Cux.) dolosus* aff.	*Culex (Cux.) maxi*	10/03	EX	*Plasmodium* sp./ pCULMAX01/MW492363
884 *	884 *	*Aedes (Och.) scapularis*	*Aedes (Och.) scapularis*	30/06	BA	*Haemoproteus (Parahaemoproteus)* sp./ hAEDSCA01/MW492364
Pool 36	887–889	*Culex (Cux.) acharistus*	*Culex (Cux.) nigripalpus*	17/03	R61	*Plasmodium* sp./ pVIOLI03/MW492365
Pool 103	634	*Mansonia (Man.) indubitans*	*Mansonia (Man.)* spp. †	17/03	R61	*Haemoproteus (Parahaemoproteus)* sp./ hMYISWA01/MW492366
	726			24/03	R113	
	798–800			10/03	L70	
Pool 119	947–954	*Culex (Cux.) coronator* complex	*Culex (Cux.)* spp. †	03/03	BA	*Plasmodium* sp./ pCULEX05/MW492367
Pool 135	461	*Culex (Cux.) coronator* complex	*Culex (Cux.)* spp. †	17/03	R61	*Plasmodium* sp./ pCULEX06/MW492368
	463					
	630–633					

# Identification of species in terms of best close match using *cox1* sequences; * specimen collected in aspirator trap; † unidentified.

**Table 3:** Landscape metrics and its percentages (in brackets) in relation to the total area for each of the eight mosquito collection sites in São Paulo Zoo.

Site	Forest Area Edge Length (m)	Forest Area (High Trees) (m^2^)		Lake (m^2^)		Edification (m^2^)		Paving (m^2^)		Bush/Sand/Grass (m^2^)		Unclassified (m^2^)	
PO	1647	12,556	(49.3)	7034	(27.6)	0	(0)	4355	(17.1)	1277	(5.0)	224	(0.9)
R69	1340	19,495	(76.6)	2295	(9.0)	0	(0)	2709	(10.6)	633	(2.5)	314	(1.2)
L70	1792	10,928	(42.9)	8247	(32.4)	0	(0)	5541	(21.8)	0	(0)	730	(2.9)
EX	1093	13,382	(52.6)	0	(0)	7438	(29.2)	4142	(16.3)	285	(1.1)	199	(0.8)
BA	1832	10,972	(43.1)	0	(0)	5348	(21.0)	5565	(21.9)	2302	(9.0)	1256	(4.9)
FM	1305	12,323	(48.4)	2006	(7.9)	220	(0.9)	6531	(25.7)	2370	(9.3)	1996	(7.8)
R113	181	24,530	(96.4)	0	(0)	0	(0)	569	(2.2)	328	(1.3)	19	(0.1)
R61	1602	14,144	(55.6)	963	(3.8)	1533	(6.0)	2432	(9.6)	6124	(24.1)	249	(1.0)

**Table 4:** Blood meal sources identified and number of engorged female mosquitoes from São Paulo Zoo.

Vertebrate Hosts		*Ae.* *scapularis*	*An.* *evansae*	*Cx.* *ameliae*	*Cx.* *chidesteri*	*Cx.* *coronator* complex	*Cx.* *declarator*	*Cx.* *habilitator*	*Cx.* *scimitar*	*Cx.* (*Mel.*) Melanoconion Section	*Cx. (Cux.)* spp.	*Ma.* *titillans*	*Ur.* *pulcherrima*
Family	Host Species												
Birds				100%		60%	50%	100%		75%	40%		
Accipitridae	*Spizaetus* *ornatos*						1 (BA)						
Anatidae	*Anser* *anser*							1 (L70)					
	*Cygnus* *atratus*			1 (R69)		1 (BA)					1 (PO)		
Ardeidae	*Ardea* *herodias*									1 (L70)			
	*Nycticorax* *nycticorax*						1 (BA)			1 (L70)	1 (PO)		
Cathartidae	*Cathartes* *melambrotus*						1 (FM)						
Cracidae	*Pipile* *jacutinga*						1 (FM)						
Gruidae	*Balearica* *regulorum*						1(FM)						
Phasianidae	*Pavo* *muticus*					1 (BA)							
Rheidae	*Rhea* *americana*									1 (R69)			
Turdidae	*Turdus* *rufiventris*					1 (R69)							
Mammals		100%	100%		100%	40%	50%		100%	25%	60%	100%	100%
Canidae	*Canis lupus* *familiaris*						1 (FM)						
Didelphidae	*Didelphis* *aurita*						1 (BA)						
Hominídea	*Homo* *sapiens*	1 (BA)	1 (R69)		1 (BA)	1(BA) 1(R61)	2 (BA) 1 (FM)		1 (R113)	1 (PO)	2 (L70) 1 (R69)	1 (EX)	1 (FM)
Total		1	1	1	1	5	10	1	1	4	5	1	1
BA, PO, R69, L70, R61, R113, EX, and FM are sampling sites as described in the Materials and Methods section.													

## Data Availability

The data presented in this study are available in Appendix A and GenBank database (https://www.ncbi.nlm.nih.gov/genbank/) (accession numbers MW492356-MW492368).

## Appendix A

**Table A1:** Climatic conditions of mosquito collection dates (DD/MM/YY).

	03/03/2015	10/03/2015	17/03/2015	24/03/2015
Minimum temperature (°C)	17.2	19.6	17.8	15.8
Maximum temperature (°C)	29.1	27.4	27.0	25.4
Average temperature (°C)	22.1	22.4	21.0	19.7
Rainfall (mm)	0	0.016 (1.6 min *)	0.012 (0.8 min *)	0

* Rainfall time.

**Table A2:** Blood meals identified according to the collection site, mosquito, and host species.

Collection Site ID	Mosquito Species	Host Species	Number Bloodmeals Identified
BA	*Aedes (Och.) scapularis*	*Homo sapiens*	1
	*Culex (Cux.) chidesteri*	*Homo sapiens*	1
	*Culex (Cux.) coronator* complex	*Cygnus atratus*	1
	*Culex (Cux.) coronator* complex	*Homo sapiens*	1
	*Culex (Cux.) coronator* complex	*Pavo muticus*	1
	*Culex (Cux.) declarator*	*Didelphis aurita*	1
	*Culex (Cux.) declarator*	*Homo sapiens*	2
	*Culex (Cux.) declarator*	*Nycticorax nycticorax*	1
	*Culex (Cux.) declarator*	*Spizaetus ornatos*	1
EX	*Mansonia (Man.) titillans*	*Homo sapiens*	1
FM	*Culex (Cux.) declarator*	*Canis lupus familiaris*	1
	*Culex (Cux.) declarator*	*Cathartes melambrotus*	1
	*Culex (Cux.) declarator*	*Homo sapiens*	1
	*Culex (Cux.) declarator*	*Pipile jacutinga*	1
	*Uranotaenia (Ura.) pulcherrima*	*Homo sapiens*	1
	*Culex (Cux.) declarator*	*Balearica regulorum*	1
L70	*Culex (Cux.) habilitator*	*Anser anser*	1
	*Culex (Mel.)* Melanoconion Section	*Ardea herodias*	1
	*Culex (Mel.)* Melanoconion Section	*Nycticorax nycticorax*	1
	*Culex (Cux.)* sp.	*Homo sapiens*	2
PO	*Culex (Cux.)* sp.	*Cygnus atratus*	1
	*Culex (Cux.)* sp.	*Nycticorax nycticorax*	1
	*Culex (Mel.)* Melanoconion Section	*Homo sapiens*	1
R61	*Culex (Cux.) coronator* complex	*Homo sapiens*	1
R69	*Anopheles (Nys.) evansae*	*Homo sapiens*	1
	*Culex (Cux.) ameliae*	*Cygnus atratus*	1
	*Culex (Cux.) coronator* complex	*Turdus rufiventris*	1
	*Culex (Cux.)* sp.	*Homo sapiens*	1
	*Culex (Mel.)* Melanoconion Section	*Rhea americana*	1
R113	*Culex (Cux.) scimitar*	*Homo sapiens*	1
